# Factors influencing quality of life and well being in metastatic prostate cancer patients

**DOI:** 10.1192/j.eurpsy.2023.1999

**Published:** 2023-07-19

**Authors:** K. Bouassida, A. Loghmari, M. Ben Othmen, G. Tlili, E. Acacha, W. Ben Abdallah, M. Kahloul, W. Hmida, M. Jaidane

**Affiliations:** 1 Urology; 2 Anesthesiology department, Sahloul teachin Hospital Sousse, Sousse, Tunisia

## Abstract

**Introduction:**

Improving health-related quality of life (HRQL) is the main goal of palliative care. It requires consensual management strategies and specific measures targeting modifiable factors that could affect the quality of life.

**Objectives:**

The aim of this study was to identify factors influencing the quality of life of patients with metastatic prostate cancer in a limited resources country.

**Methods:**

This is a retrospective and analytical study enrolling all patients with metastatic prostate cancer who were managed at medical oncology and urology consultations of two Tunisian teaching hospital. HRQL was measured using UCLA prostate cancer index and SF-36 SCALE. The influence of demographic and medical characteristics on HRQL was determined using t tests and analysis of variance, with Tukey’s correction for multiple comparisons.

Multivariate linear regression was used to determine independent predictor

**Results:**

This study enrolled 244 patients. The mean age was 72 years. The strongest determinants of overall HRQL after univariate analysis were: increased age (p = 0.006), lower income (p = 0.009), sexual function problems (p = 0.004), urinary function problems (p = 0.002) and symptoms such as pain (p = 0.001) and asthenia (p = 0.001).

**Conclusions:**

Age, income, sexual and urinary functions are important determinants of HRQL in patients with metastatic prostate cancer that may require specific interventions.

**Disclosure of Interest:**

None Declared

